# Acute myocardial infarction with non-obstructive coronary artery disease due to plaque erosion treated with balloon-occluded thrombolysis

**DOI:** 10.1093/ehjcr/ytae461

**Published:** 2024-08-27

**Authors:** Pitt O Lim

**Affiliations:** Department of Cardiology, Atkinson-Morley Wing, St George’s University Hospitals NHS Foundation Trust, Blackshaw Road, Tooting, London SW17 0QT, UK

A 55-year-old man presented with an ST-elevation myocardial infarction (STEMI) 4.5 h from symptomatic onset. Thrombectomy was performed to his occluded right coronary artery (RCA) (*Panels A–C*, Supplementary material online, *Videos S1* and *S2*), which restored flow to the RCA crux. However, the outflow vessels were thrombosed and ST-elevation persisted with pain. Given the widespread thrombi, the mid-RCA was balloon occluded and a half-dose of weight-adjusted tenecteplase (Boehringer Ingelheim, Germany) was delivered via a microcatheter. After 10 min (*Panel D*, Supplementary material online, *Video S3*), these devices were removed, and the remaining tenecteplase was given directly through the guide catheter. The subsequent flow in RCA improved (*Panel E*, Supplementary material online, *Video S4*). Following 3 days of subcutaneous anti-Xa anticoagulation, the patient underwent an optical coherence tomography (OCT). There was a mild-to-moderate segment of disease immediately beyond the site of occlusion, but with unimpeded flow distally (*Panel F*, Supplementary material online, *Video S5*). There were no breaches in the vascular endothelium continuity to suggest a plaque rupture on OCT (*Panel G*). Hence, stenting was not deemed to be required. The electrocardiographic R-wave remained without a Q-wave (*Panel H*), and left ventricular angiogram demonstrated intact inferior wall contractility (*Panel I*, Supplementary material online, *Video S6*). Taken together, these imply viable myocardium and his echocardiogram showed a normal heart. He was discharged home the same day post-procedure.

**Figure:**
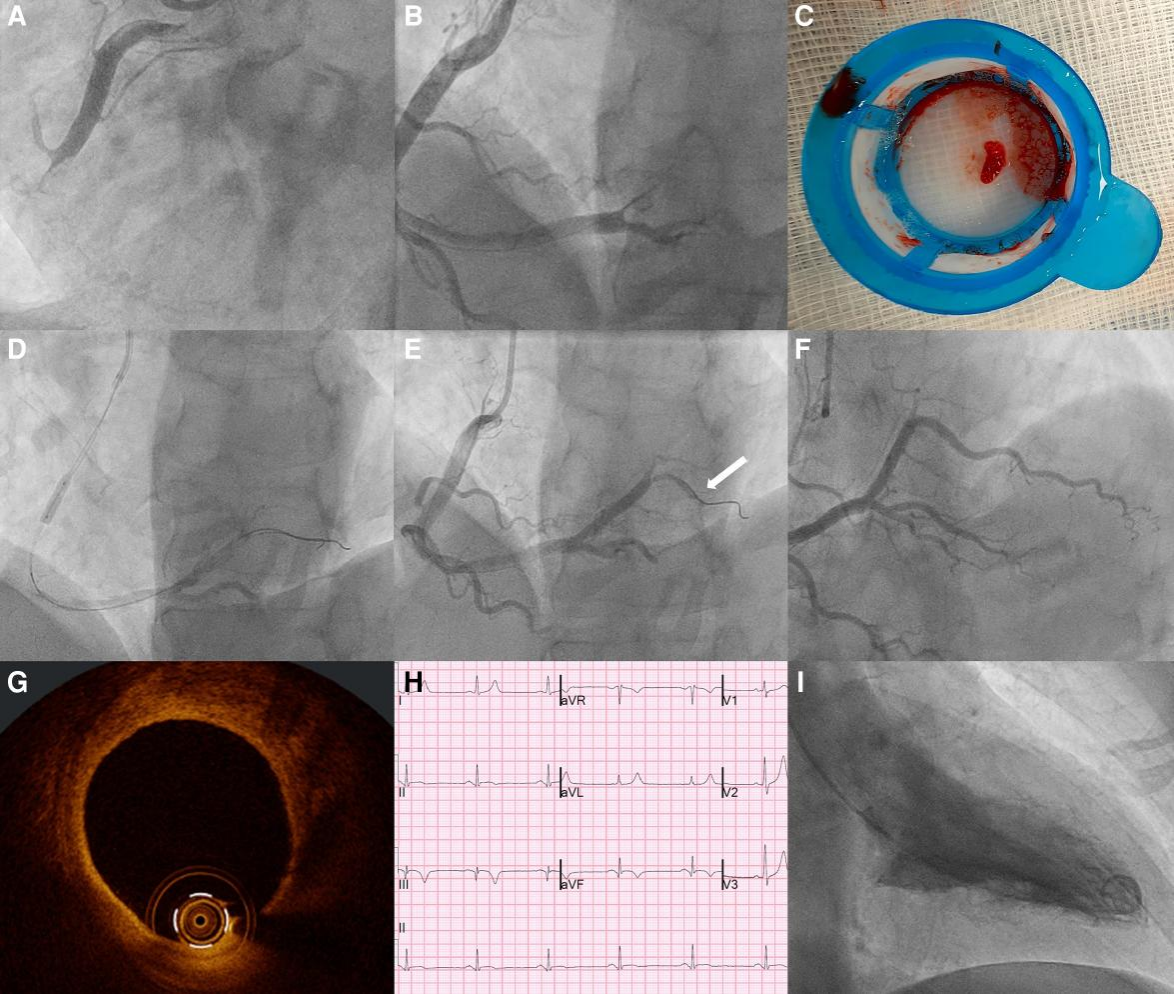
**Right coronary artery thrombotic occlusion, thrombectomy and intracoronary thrombolysis** Coronary angiogram images show an occluded mid-right coronary artery (*Panel A*); immediately post-thrombectomy with flow down to the crux (*Panel B*); and extracted thrombus (*Panel C*). Balloon occlusion with direct thrombolysis, the artery was opacified with contrast using a dual-lumen microcatheter (*Panel D*). Second half-dose thrombolytic agent was given with partial dissolution of distal thrombi (*Panel E*; arrow); Day 4 coronary angiogram shows complete clearance of clots with unrestricted flow (*Panel F*); optical coherence tomography finds uninterrupted endothelium with thin-cap fibroatheromatous (TCFA) plaque (*Panel G*); electrocardiogram reveals persistent R-wave in the inferior leads (*Panel H*); and together with left ventricular angiogram in systole that demonstrates inferior wall contractility (*Panel I*), are consistent with preserved myocardial function.

ST-elevation myocardial infarction is primarily a thrombotic event, often without a significant underlying coronary artery stenosis. Myocardial salvage is time sensitive. It also depends on minimizing microvascular obstruction by embolizing clots or showering of atherosclerotic debris, which can be exacerbated by stenting. Thus, the new focus is in aetiopathologically based STEMI management in differentiating between plaque rupture, erosion, and calcium protrusion, as well as coronary dissection and vasospasm. Finally, adjunctive glycoprotein IIb/IIIa inhibitor is often used in such a case, when intracoronary thrombolysis may be better; the former prevents new clots, whereas the latter dissolves existing thrombi.

## Supplementary Material

ytae461_Supplementary_Data

## Data Availability

The data underlying this article can be shared on reasonable request to the corresponding author.

## Notes

**Consent:** The author confirms that written consent for submission and publication of this cardiovascular flashlight including images and associated text has been obtained from the patient.

**Funding:** None declared.

